# ECONOMIC HARDSHIP INDICATORS ARE DIFFERENTIALLY ASSOCIATED WITH ADVERSE HEALTH OUTCOMES IN OLDER ADULTS

**DOI:** 10.1093/geroni/igz038.461

**Published:** 2019-11-08

**Authors:** Loretta Anderson

**Affiliations:** Johns Hopkins Bloomberg School of Public Health, Baltimore, Maryland, United States

## Abstract

Previous studies have shown that higher levels of economic hardship in older adults is associated with increased odds of adverse health outcomes such as insomnia, anxiety, and depressive symptoms. The objective of this study was to determine if there was a differential association between individual measures of economic hardship and aforementioned adverse health outcomes. Cross-sectional analysis was conducted using data from the 2012 National Health and Aging Trends Study (NHATS). Logistic models were developed to assess the association between each of the four measures of economic hardship and three previously reported adverse health outcomes. Participants were asked if in the last month they did not have enough money for food, utility bills, mortgage/rent, or medical bills/prescription drugs. Measures of adverse health outcomes were symptoms of depression, anxiety, and insomnia. There were 7,075 community dwelling older adults aged 65 and older in the 2012 NHATS data. Results indicated that those who skipped meals were more likely to have depression, anxiety, and insomnia symptoms than those who did not skip meals. After adjusting for race, age, gender, education, and comorbid health conditions, skipping meals was associated with depression (OR=4.11, p<.000), anxiety (OR=2.81, p<.01), and insomnia (OR=2.16, p<.05). These results were higher and more statistically significant than the other measures of economic hardship. These findings are relevant to population-based efforts of nutrition interventions to improve quality of life in aging populations.

